# Morphological and Phenological Diversity of Pod Corn (*Zea mays* Var. *Tunicata*) from Mexico and Its Functional Traits Under Contrasting Environments

**DOI:** 10.3390/plants15020280

**Published:** 2026-01-16

**Authors:** Teresa Romero-Cortes, Raymundo Lucio Vázquez Mejía, José Esteban Aparicio-Burgos, Martin Peralta-Gil, María Magdalena Armendáriz-Ontiveros, Mario A. Morales-Ovando, Jaime Alioscha Cuervo-Parra

**Affiliations:** 1 Escuela Superior de Apan, Universidad Autónoma del Estado de Hidalgo, Carretera Apan-Calpulalpan, Km 8, Chimalpa Tlalayote, Apan 43920, Hidalgo, Mexico; romero@uaeh.edu.mx (T.R.-C.); jose_aparicio@uaeh.edu.mx (J.E.A.-B.); martin_peralta10391@uaeh.edu.mx (M.P.-G.); maria_armendariz@uaeh.edu.mx (M.M.A.-O.); 2 Escuela Superior de Ciudad Sahagún, Universidad Autónoma del Estado de Hidalgo, Carretera Ciudad Sahagún-Otumba s/n, Zona Industrial, Sahagun City 43990, Hidalgo, Mexico; 3 El Terregal de Ray, San Dionisio Yauhquemehcan 90457, Tlaxcala, Mexico; rayo2882@yahoo.com.mx; 4 Sede Acapetahua, Universidad de Ciencias y Artes de Chiapas, Calle Central Norte s/n Entre 4ª y 5ª Norte, Acapetahua 30580, Chiapas, Mexico; mario.morales@unicach.mx

**Keywords:** tunicate maize, leafy glume, phenotypic plasticity, ear development, landraces, Mexico, proximate composition

## Abstract

Pod corn (*Zea mays* var. *tunicata*) bears leafy glumes that enclose kernels, resembling a partial reversion to wild-forms, yet remains poorly characterized in situ in Mexico. We evaluated Mexican accessions at two contrasting locations to quantify morphological/phenological diversity and to assess functional traits via proximate kernel composition. Standard descriptors captured variation in plant architecture, tassel/ear traits (including glume length), and reproductive timing. Accessions showed strong plasticity and significant accession × environment effects on ear morphology and maturation. Grain yield ranged from 6.32 to 10.78 t ha^−1^, with peak values comparable to commercial hybrids and above-typical yields reported for native Mexican races (2.7–6.6 t ha^−1^). Proximate analysis showed that milling with the tunic increased moisture/ash (up to 3.07% vs. 1.80% in dehulled grain), tended to lower fat and protein, and yielded lower crude fiber than dehulled samples (0.78–0.96% vs. 1.59–1.77%); protein varied widely (1.05–6.64%). Thus, the tunic modulates elemental composition, informing processing choices (with vs. without tunic). Our results document a spectrum of morphotypes and highlight developmental diversity and field adaptability. The observed accession × environment responses provide a practical baseline for comparisons with native and improved varieties, and help guide product development strategies. Collectively, these data underscore the high productive potential of pod corn (up to 10.78 t ha^−1^ under optimal management) and show that including the tunic substantially alters proximate composition, establishing a quantitative foundation for genetic improvement and food applications. Overall, pod corn’s distinctive ear morphology and context-dependent composition reinforce its value for conservation, developmental genetics, and low-input systems.

## 1. Introduction

Pod corn (*Zea mays* var. *tunicata* A. St. Hil.) is a traditional maize native to the Mexican states of Tlaxcala, Durango, and Chihuahua, where it retains its distinctive morphological traits [1]. In San Juan Ixtenco, Tlaxcala, considered the last stronghold of the Otomi community, farming families actively conserve this landrace, recognized as one of the oldest and most resilient in the region [2]. In this municipality, ears from tunicate plants are used for decorative, medicinal, ceremonial, and culinary purposes, as well as for animal feed and specialty food for human consumption [3,4]. Maize is monoecious, producing separate male (tassel) and female (ear) inflorescences. These structures arise from primordial staminate and gynoecial cells; the gynoecium develops in the ear and the staminate tissue in the tassel [5]. Under normal development, gynoecial primordia in the tassel and staminate primordia in the ear are eliminated via programmed cell death (PCD), preventing abnormal bisexual or sex-reversed inflorescences [6,7]. Nevertheless, multiple mutants affecting sexual organ identity have been described, resulting in opposite-sex or bisexual inflorescences due to perturbations in PCD-regulatory genes [8]. In addition, plant hormones such as gibberellic acid modulate floral sex determination and organ abortion in maize [9].

In maize, *tasselseed* (*ts*) genes drive sex conversion in the inflorescences. Mutations in *ts4*, *ts5*, and *ts6* confer varying degrees of tassel bisexuality, whereas *ts1* and *ts2* can produce fully pistillate (female) tassels [7,9]. The *ts2* locus encodes a factor required for gynoecium abortion [10]. Developmental analyses indicate that *ts4* and *ts6* mutations disrupt or delay the differentiation of specific spikelet meristems in both the tassel and ear and may inhibit pistil development in tassels [7]. Such mutations have been documented in tunicate maize [4,11]. A hallmark of pod corn is the codominant *Tunicate* (*Tu*) mutation, which enlarges the leaf-like, non-reproductive organs of male and female inflorescences and produces a husk-like tunic around each kernel [12,13]. In tassels, interaction with the *ts* activity can permit the formation of functional pistils bearing silks and, ultimately, grains [9]. Ears exhibit pedicellate and sessile kernels enveloped by leafy glumes [14]. Molecular work has shown that *Tu* corresponds to a MADS-box developmental regulator (*ZMM19*), with altered expression patterns characteristic of the MADS-box gene family [13,15], and this is further supported by expression studies in pod corn [11]. Wild-type ears lacking the *Tu* alteration in *ZMM19* do not form tunicate kernels [13].

Historically, these unusual traits led to the proposal that pod corn represented a “missing link” between modern maize and its wild relatives, the teosintes [16], from which maize was domesticated in Mesoamerica [17]. However, subsequent molecular and developmental evidence clarified the genetic basis of the tunicate condition and demonstrated that pod corn is not ancestral to modern cultivated maize [11,13,14,15]. Interactions between *ts* and *Tu* alleles shape tassel and ear morphology, yielding phenotypic classes that include wild-type, *Tu* heterozygotes, and *Tu* homozygotes [7,11]. Despite international interest, pod corn in Mexico has received limited scientific attention and remains relatively unfamiliar to the general public [3]. Interest is resurging in both scientific and outreach venues [18]. Here, we characterize the morphology, assess the phenology, and determine the proximate composition of tunicate maize accessions from San Juan Ixtenco, Tlaxcala, evaluated at two experimental sites in Mexico.

While recent molecular studies have continued to elucidate the genetic basis and pleiotropic effects of the *Tunicate 1* (*Tu1*/*ZMM19*) locus, contemporary field-based agronomic and phenological characterizations of tunicate maize remain limited [19]. Notably, a recent study in traditional polyculture agroecosystems of San Juan Ixtenco profiled functional groups of culturable rhizobacteria associated with tunicate maize at tasseling and maturity/senescence stages, underscoring both the biocultural relevance of this landrace and the need for integrative agronomic evaluations [20]. Accordingly, the present work provides a phenotypic baseline for phenotype-derived seed lots and does not attempt to estimate genetic parameters (e.g., heritability, genetic variance, genetic gain) or infer inheritance patterns, which would require structured progeny families (half-sib/full-sib) and/or genotyping.

## 2. Results and Discussion

For clarity, we first report the patterns directly observed and quantified in our field trials (Tables/Figures). Any statements regarding domestication history, teosinte introgression, or trait erosion are literature-based interpretations and are explicitly framed as hypotheses, because our study did not include genotyping or controlled-cross designs.

**Figure 1:**
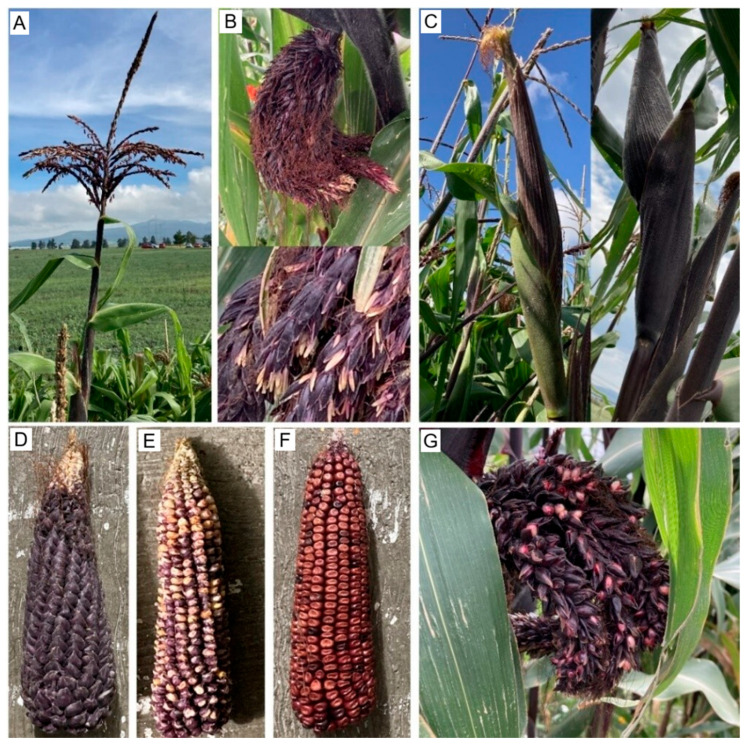
Reproductive phenotypes observed in tunicate maize. (**A**) Normal staminate tassel. (**B**). Hermaphroditic tassel—bearing stigmas and pistillate florets alongside dehiscent anthers. (**C**) Tassel-less, “female” plants with apical ears at different developmental stages. (**D**) Tunicate ear. (**E**) Semi-tunicate ear. (**F**) Ear lacking a tunic. (**G**) Hermaphroditic tassel with grain set.

In this study, morphotype terminology was defined as follows. Tp1 plants showed a normal tassel and typically produced one or two large ears (often E1–E3), Tp2 plants had a hermaphroditic tassel capable of setting grain and commonly produced ears with a tunic (E2–E3), Tp3 plants lacked a tassel because an apical ear replaced the tassel (Figure 1C) and their degree of tunication depended on the pollen source, and Tp4 plants developed multiple ear-bearing nodes and were associated with smaller ears classified as E4 (small tunicate) or E5 (small non-tunicate); ear morphotypes were classified as E1 (non-tunicate), E2 (strongly tunicate), E3 (partially tunicate), E4 (small tunicate), and E5 (small non-tunicate), and treatments (T1–T4) corresponded to phenotype-defined seed lots.

### 2.1. Preliminary Experimental Plots

The results of this study show that across the preliminary plots, most vegetative traits differed significantly (Table 1). Mean plant height in P1 reached 264.24 cm, slightly exceeding the 257.89 cm recorded in P2. This contrast may reflect combined differences in site conditions and the fertilization regimes applied to P1 and P2, see Materials and Methods, Section 3.2); therefore, it is interpreted descriptively rather than as a causal estimate of fertilization effects. Previous studies have shown that integrated fertilization, combining microbial inoculants, mineral fertilizers, and organic amendments, can increase maize height [21,22,23,24,25]. In line with evidence, the more diverse formulation and higher input levels used in P1 may account for the greater plant heights observed.

Significant differences in tiller number per plant were observed between treatments; P1 averaged 2.99 tillers per plant, whereas P2 averaged 2.56 tillers per plant. By contrast, stem diameter did not differ between plots (2.91 cm in P1 vs. 2.90 cm in P2), aligning with the 2.9 cm reported for Chalqueño race corn [25] and exceeding the 2.06 cm documented for Kculli [24]. Grain yield likewise showed no significant difference, with 12.23 and 11.91 t ha^−1^ for P1 and P2, respectively. Assessment of phenological traits revealed additional variables with significant differences beyond the initial set; details are provided in Table 2.

Grain yields in both tunicate maize plots exceeded previously reported values for Kculli and Chalqueño (6.19–6.57 t ha^−1^) cultivated under comparable fertilization regimes [24,25]. This advantage likely reflects the pronounced tillering capacity of this race (≈1–6 tillers per plant), a trait shared with pod corn and both annual and perennial teosintes [17,26,27]. Moreover, tillers commonly bear ears, and those with hermaphroditic tassels can set grain, further increasing stand-level yield.

Tiller production from basal nodes is a characteristic feature of grasses [28]. In maize, tillers are often concentrated in border rows, where reduced competition and greater space and light availability favor the development of secondary stems [29]. In contrast, pod corn in plots P1 and P2 produced tillers uniformly across all rows and replicates. Early-emerging tillers typically set viable ears and grain, whereas late-emerging tillers generally bear ears with little or no grain [29,30]. In our case, tillers emerged early and progressed synchronously to reproduction, yielding viable ears. Tiller expression is modulated by genotype and environment-race-specific genetics, and lower planting densities increase tillering [30,31]. Although density may have contributed, tillers were already present when three seedlings per hill were still intact before thinning, indicating a strong genetic predisposition. This tendency could reflect (among other factors) a working hypothesis of introgression from wild teosintes sympatric with pod corn in the region [4]; however, this scenario cannot be tested with our dataset and would require genotyping and/or controlled crosses.

Complementary evidence from traditional highland agroecosystems indicates that tunicate maize hosts diverse culturable rhizobacteria with plant growth-promoting activities across tasseling and maturity/senescence stages, which may contribute to plant vigor under rainfed polyculture conditions [20].

Mean ear number per plant was 2.59 in P1 and 2.06 in P2. Beyond ear number, tassel phenotypes clustered into three classes with plot-specific frequencies (Table 2). (i) Normal tassels occurred on 40.33% and 59.67% of stems (Figure 1A), corresponding to 30.09% (P1) and 51.00% (P2) of plants. (ii) Abnormal reproductive structures attributable to the *ts* and *Tu* mutations [9,13], yielding hermaphroditic tassels and tunicate ears [11,14], were recorded in a mean of 91.33 and 56.00 plants, representing 68.16% (P1) and 47.86% (P2); these structures were typically filled with grain, causing the tassel to overdevelop and droop (Figure 1B). (iii) Plants lacking a tassel (“female plants”) were rare, 2.33 (1.73%) in P1 and 1.33 (1.13%) in P2 (Figure 1C). The physiological basis of tassel loss with an apical ear remains unclear, but it may be influenced by environmental cues and the presence of additional stems [32]; management practices can also modulate its incidence [29]. Consistent with these tassel variants, tunicate plants exhibited three ear morphotypes (Figure 1D–F) and grain set within hermaphroditic tassels (Figure 1G), in line with previous reports for this race [4,11].

### 2.2. Morpho-Phenological Variation in Tunicate Maize (2023–2024 Cycles)

The results of this study show that using data from plot P1 across the 2023–2024 cycles, four plant morphotypes were defined by phenological traits (Tp1–Tp4), and five ear morphotypes (E1–E5) were distinguished within each treatment. Because treatments were phenotype-derived composite seed lots produced under open pollination, the differences reported among T1–T4 are interpreted as phenotypic divergence among seed lots under the evaluated conditions rather than estimates of inheritance patterns, segregation ratios, or population genetic parameters.

Percentages/frequencies of plant and ear morphotypes reported below are derived from the corresponding counts per treatment (*n* = 3 replicates) and were tested statistically using the models described in Section 3.5; when overall effects were significant, Tukey-adjusted comparisons were applied (*p* < 0.05).

**Figure 2:**
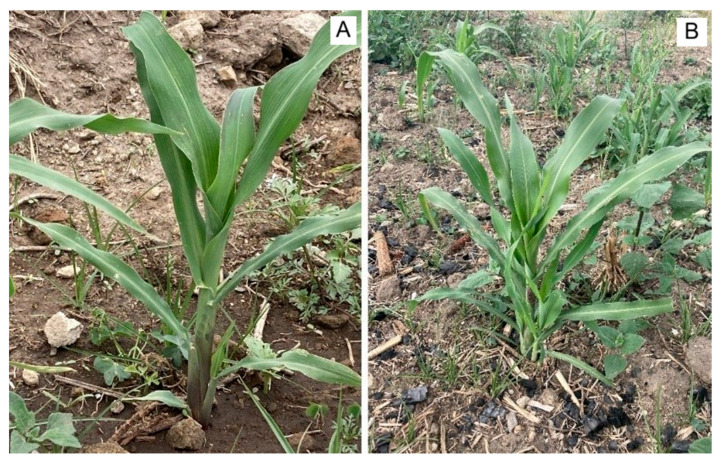
Tunicate maize at representative vegetative stages. (**A**) V8 plant with one additional tiller. (**B**) V11 plant with two developing tillers.

During early vegetative growth, no morphological differences were evident within treatments. The first additional tiller appeared at V8, ≈30–35 days after emergence (Figure 2), consistent with reports that tiller initiation typically occurs near V7 in other maize races [33]. Divergence among morphotypes becomes apparent at the onset of reproduction. In this sense, Tp1 (“normal” habit) plants developed a conventional staminate tassel (Figure 1A), and produced one or two large ears lacking tunic, 12.0–19.8 cm in length (mean = 16.03 length, SE = 2.03, *n* = 50), comparable to commercial maize grown in the Highlands of Mexico [34,35].

Tp2 (hermaphroditic tassel): Tassels bore pistillate structures within glumes alongside functional stamens; after fertilization, these produced grain and often drooped due to weight, frequently with a complete or partial tunic (Figure 1G), as described for tunicate maize [11,13]. A tunicate ear was present in 49.24 ± 3.52% of cases, and a sparse tunic in 13.04 ± 0.25%. This phenotype reflects a codominant *Tu* mutation that disrupts transcriptional control in spikelet development; two tandem copies on chromosome 4 and altered regulatory regions underlie tunicate expression, with a clear gene-dosage effect [12,13,15].

Tp3 (apical ear replacing tassel): Rare plants lacked a tassel and instead formed an apical ear (Figure 1C), with tunic presence depending on pollen source (Tp1 → non-tunic; Tp2 → tunicate). This morphotype was observed only in T1 at very low frequency (1.49 ± 0.001%). Maize is monoecious, with early bisexual primordia undergoing sex-specific abortion, gynoecium in the tassel, stamens in the ear, through genetic and hormonal control [36,37,38,39]. Mutations in regulators of programmed cell death can generate opposite sexual structures or bisexuality [8,33]. In the Tp3 morphotype, the pattern is reversed: male reproductive structures abort, and female organs are retained on both tillers and the main stem. The resulting phenotype ranges from complete tassel to ear conversion, often yielding huskless ears exposed to the environment, to partial conversions in which only a segment of the tassel is replaced by ear tissue that sets kernels [29]. Environmental factors may also induce apical ears [40]. In pod corn, the dominant *Tu* mutation promotes sexual inversion in the tassel and husk formation at the apex, protecting kernels [11,13,32].

**Figure 3:**
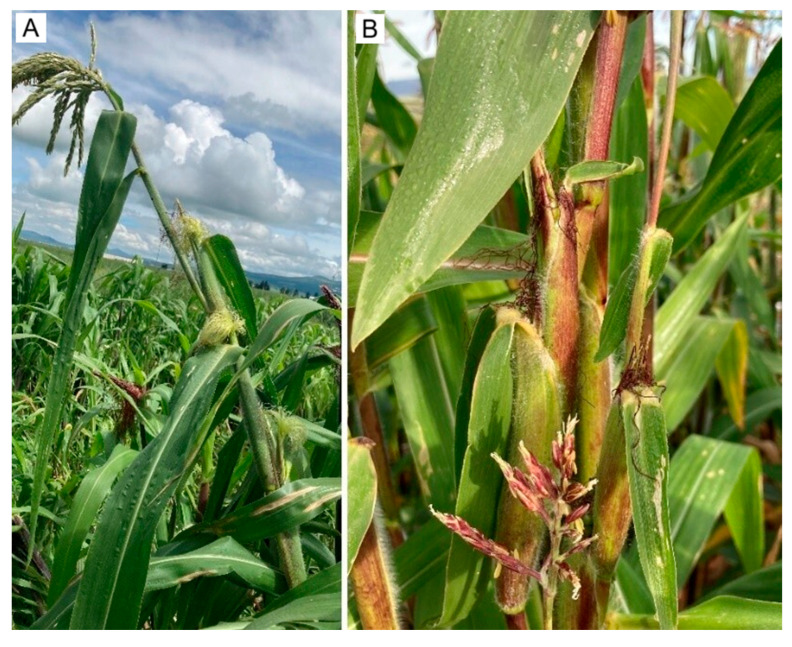
Comparative ear proliferation in pod corn and teosinte. (**A**) Tunicate maize (Tp4 morphotype) with three ear-bearing nodes developing synchronously. (**B**) Annual teosinte (*Z. mays* subsp. *mexicana*) showing multiple ears per node.

Tp4 (multi-ear nodes): Recorded only in P1 during 2023–2024 and absent from preliminary plots, Tp4 plants displayed normal vegetative growth but, at sexual maturity, developed a typical tassel plus multiple small ears (1–5 per node) across several stem nodes (Figure 3A), resembling annual teosintes *Zea mays* ssp. *mexicana* [17] (Figure 3B).

In rare instances, commercial maize varieties can set multiple ears at a single node on the main stem [41]. This expression is generally atypical and reflects an integrated response to biotic and abiotic factors together with genetic background and management [42,43,44]. Contributing drives include loss of dominance by the primary ear, surplus resources (nutrients), and abundant sunlight within the canopy [32,45]. The condition, termed “bouquet ears” [41] or “shank ears” [46], arises from activation of axillary meristems at stem nodes, typically initiating in early vegetative stages (V4–V6) and sometimes continuing through tasseling/silking (VT-R1) [47,48]. Although each node can initiate an ear [49], the primary ear usually suppresses additional ears at lower nodes [29]. When secondary ears do form, they often remain stunted or abnormal, potentially reducing yield if prevalent [50]. In the Tp4 morphotype, however, multiple ears within a node were consistently small yet discrete and showed normal grain fill, indicating synchronous initiation; this contrasts with many commercial genotypes, where floral asynchrony commonly leaves secondary ears barren [40,50].

Across treatments, Tp4 occurrence was 0.00% (T1), 1.24% (T2), 1.48% (T3), and 15.44% (T4). Among Tp4 plants in T2–T4, 73% of ears were tunicate, and 27% lacked a tunic. The *Tu* mutation is absent in teosinte, likely arising post-domestication [11,51], but environmental stress, genetic background, sparse stands, and silk damage can modulate ear proliferation and fertility [29,52]. Moreover, repeated clipping of ear silks by beetles or butterflies can prevent pollination on the primary ear and induce the growth of secondary ears, which are usually sterile [41].

**Figure 4:**
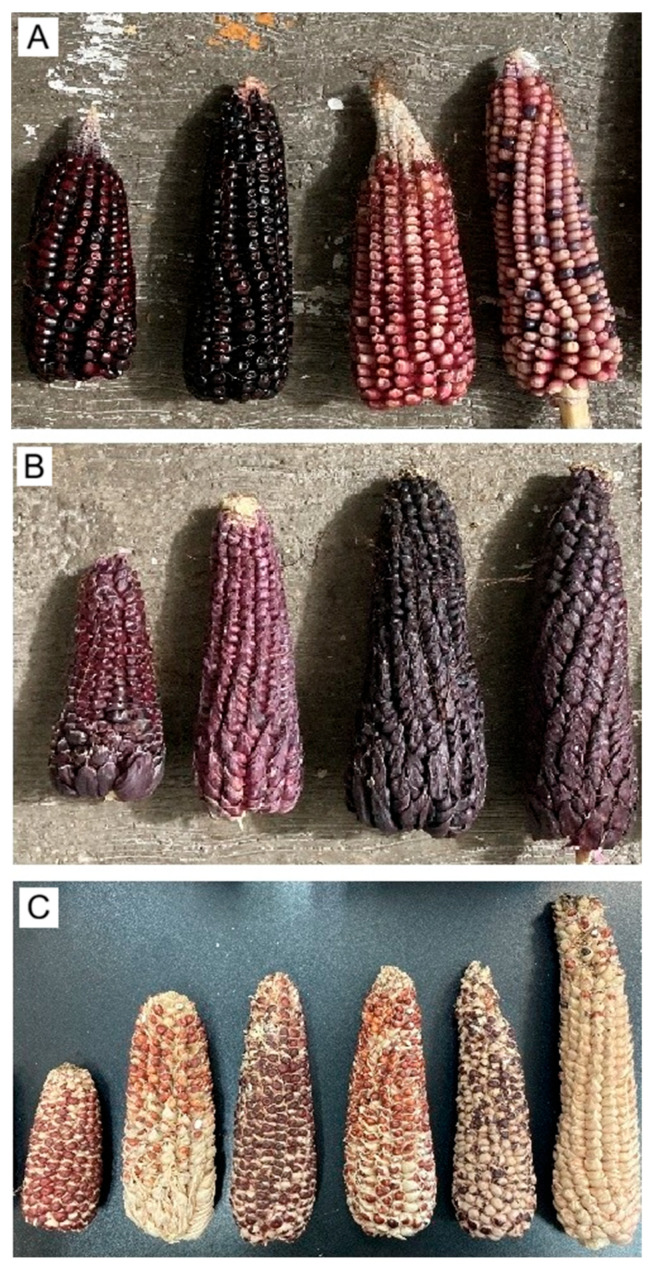
Ear morphotypes in tunicate maize (E1–E3). (**A**) E1 non-tunicate ears lacking a tunic around the kernels. (**B**) E2 strongly tunicate ears with a conspicuous tunic enveloping each kernel. (**C**) E3 partially/sparsely tunicate ears with variable, often basal, coverage.

Regarding ear type across treatments, E1 ears lacked a tunic on individual grains (Figure 4A), corresponding to the typical ear found in commercial maize worldwide [24,53]. Ear length ranged from 12.0 to 19.8 cm (mean = 16.03 cm, SE = 2.03, *n* = 50), and E1 occurred in all four treatments. These values align with reports for other commercial varieties grown in the High Valleys of Mexico, such as Chalqueño (9.57–11.26 cm) and Kculli (16.3–18.95 cm) [24,25]. By contrast, E2 ears exhibited a well-developed tunic around each grain (Figure 4B) and measured 10.2–23.5 cm (mean = 17.37 cm, SE = 2.18, *n* = 50). Floral bracts (glumes) enclosing individual grains in pod corn cobs have been documented previously [3,54].

**Figure 5:**
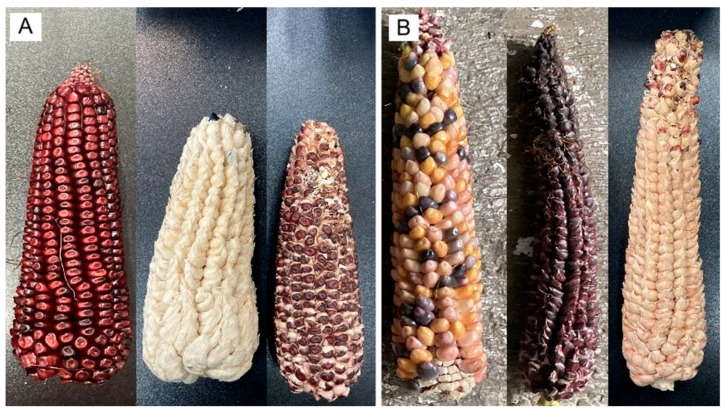
Ear shape classes in tunicate maize across E1–E3 morphotypes. (**A**) Conical. (**B**) Conical–cylindrical.

E3 ears exhibited a sparse tunic; kernels were covered by a thin tunic over the entire surface or only at the basal portion, while the media and apical regions were typically exposed (Figure 4C). Ear length ranged from 6.4 to 23.3 cm (mean = 15.72 cm, SE = 4.73, *n* = 40). This phenotype agrees with descriptions of pod corn from San Juan Ixtenco, Tlaxcala [4]. Across the two-year cultivation period and four treatments, ear shape in E1, E2, and E3 was predominantly conical, with conical–cylindrical forms only rarely observed (Figure 5). Evidence from crossbreeding experiments indicates that the tunicate trait involves at least two separable genetic factors [15]. The inheritance of a single component produces markedly smaller, less conspicuous glumes, whereas plants carrying both components develop more prominent glumes [13].

**Figure 6:**
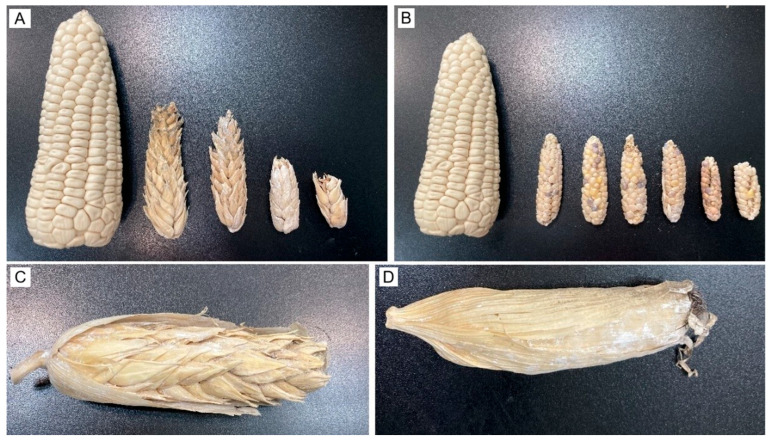
Ear phenotypes in the Tp4 morphotype. (**A**) E4 strongly tunicate kernels. (**B**) E5 non-tunicate kernels. An ear of the Cacahuacintle landrace (18 cm) is included for size reference in (**A**,**B**). (**C**) Partial husk coverage (basal only), with the apex exposed. (**D**) Complete husk enclosure.

E4 and E5 ear morphotypes were observed exclusively in Tp4 plants. To our knowledge, these alternative morphotypes have not been documented previously [11,13]. Both ear types were smaller than E1–E3; E4 measured 5.5–12.5 cm (mean = 9.97 cm, SE = 1.74, *n* = 25) and E5 measured 5.5–12.4 cm (mean = 9.20 cm, SE = 2.20, *n* = 25). Phenotypically, E4 kernels exhibited a conspicuous tunic (Figure 6A), whereas E5 kernels were non-tunicate (Figure 6B). In both morphotypes, husk coverage varied from partial, the apical portion exposed (Figure 6C), to complete enclosure (Figure 6D). Given their low frequency and the absence of structured progeny testing, E4–E5 are treated here as descriptive phenotypes; we do not infer segregation or underlying allele frequencies from their occurrence.

Across the ear variants observed in this study, including sparsely and weakly tunicate forms, the most archaic types are those with conspicuous tunicate floral bracts (glumes) enveloping the kernels [3]. Repeated crossing and hybridization of pod corn with commercial maize cultivated in the same region has been proposed to eroded the tunicate trait over time, culminating in modern maize with non-tunicate kernels [3,4]. In our study, we directly observed this continuum: plants bearing ears with abundant tunics (Figure 4B); ears with a sparse tunic, often absent in the central portion (Figure 4C); and ears entirely lacking tunicate structures (Figure 4A), together with a normal tassel lacking pistils (Figure 1A). These patterns are compatible with (but do not demonstrate) pollen-mediated gene flow among pod corn, its wild relative teosinte, and local landraces [3,55], and are therefore presented here as a hypothesis. In Tp4 plants from treatments T2–T4 in the P1 plot, we additionally observed synchronous formation of several small ears at successive nodes on the main stem (Figure 3A) and compact ears (Figure 6) with morphologies resembling teosinte-like phenotypes described in the literature [55,56]. We treat this resemblance as descriptive and do not infer an evolutionary trajectory from our field observations alone.

These observations are consistent with the provenance of the seed lot, San Juan Ixtenco, Tlaxcala, from which the original pod corn samples were obtained [4]. Moreover, the E4 and E5 ear morphotypes resemble the “protocob” described for natural maize × teosinte crosses in the Ciénaga region, Jalisco, Mexico, characterized by reduced ear size, few kernel rows, and multiple ears per node [56]. A key distinction is that only the E4 morphotype carries the *Tu* mutation, which causes the glumes to completely enclose the kernels [11]. Alternatively, the appearance of multiple ears per node in Tp4 plants may reflect environmental stress (e.g., cold), genetic background, low seeding rate, and/or damage to the primary ear [29].

In E4 ears, the tunicate trait is attributed to a chromosomal rearrangement at the *Tu* locus, likely a large inversion associated with transposition of a mutator-type transposon and duplication of two genes at the breakpoint, yielding the characteristic phenotype in which glumes fully enclose the grains [11]. Short-husk ears observed in some Tp4 plants may reflect acute stress (e.g., heat, drought, sudden cold, high winds, storms) and/or genetic factors [57]. Local environmental conditions at the experimental site may also have contributed [58].

### 2.3. Vegetative and Reproductive Dependent Variables in Maize

#### 2.3.1. Vegetative Dependent Variables

The results of this study show that across the 2023–2024 growing seasons, the response variables measured during the vegetative stage of pod corn differed significantly among treatments (*p* < 0.05; Table 3). Because no year effect was detected (2023 vs. 2024; all year terms and year × treatment interactions were non-significant, *p* > 0.05), data were pooled and analyzed as two-year means. Likewise, as no significant differences were found among plant morphotypes (Tp1, Tp2, Tp3, and Tp4) within treatments for either vegetative or reproductive variables (all morphotype terms were non-significant, *p* > 0.05), values for each variable were averaged within each treatment.

Significant treatment effects were observed for plant height (main stem and tillers), number of tillers per plant, and stalk diameter (main stem and tillers) (Table 3). For main stem height, most treatments differed, with T2 plants reaching the greatest mean height (250.74 cm). These values fall within the range reported for native maize for diverse regions of Mexico (180–300 cm) [59,60]. T3 and T1 followed with comparable heights (210.61 and 210.41 cm, respectively), consistent with reports for hybrids from crosses between improved lines and native maize in the High Valleys (180–230 cm) [61]. On the other hand, T4 plants were the shortest (187.25 cm); the reduced stature may be associated with the higher frequency of hermaphroditic tassels that bend downward by the grain weight [62]. Depending on the zygosity of the *Tu* mutation in tunicate maize (heterozygous vs. homozygous), additional phenotypes may arise in tassels and ears, such as increased branching, the development of lower ear florets, and seed set in tassels [14,16], which increase tassel mass and can thereby limit plant height

Tiller height mirrored the pattern observed for main-stem height across treatments (Table 3). The tallest tillers occurred in T2 (241.55 cm), whereas T1 and T3 were statistically similar (192.24 and 192.12 cm, respectively), and T4 showed the shortest tillers (168.66 cm). These values exceed those reported for annual teosinte (93.00–176.72 cm) and perennial teosinte (110.33–137.88 cm) grown without fertilization in the same region [17]. Main stems were consistently taller than tillers in all treatments. In all morphotypes, tillers initiated near the V7 stage (≈35–45 days after germination) [33] and, because they emerge after the parent stem, compete at a resource disadvantage that limits elongation [63]. Lower planting density can also promote tillering by increasing radiation interception per plant [64,65], which is consistent with the management conditions of this study. Genetic factors may further contribute [57]; the production of basal secondary shoots is shared with other native maize from Mexico’s High Valley [66,67], and gene flow with teosinte, common where creole maize coexists with wild populations, may also influence tillering [3,68].

Tiller number varied among treatments (Table 3). T1 and T3 produced the highest averages (1.95 and 1.96 tillers, respectively), followed by T2 (1.76), whereas T4 had the lowest mean (1.50). In maize, tillering is favored under reduced planting density and is strongly genotype-dependent; greater tillering enhances interception of solar radiation [69] through the increased leaf area per plant [70]. Early-season temperature drops, together with low stand density and higher irradiance, further promote tiller initiation [30]. This window coincides with the onset of tillering observed in the tunicate corn evaluated here and in previous reports [33]. Under rainfed, low-density conditions, typical values range from 1 to 4 tillers per plant [65], whereas under irrigation, 7.6 to 8.3 tillers per plant have been reported [64]. Our observations across pod corn treatments (range: 1 to 6; most plants with 1 to 3 tillers); are consistent with these ranges. By contrast, maize’s wild relatives exhibit greater plasticity: annual teosinte shows 1 to 15 tillers per plant [26,27] and perennial teosinte 15 to 18 [17].

Main-stem diameter differed among treatments (Table 3). T2 plants showed the greatest mean diameter (2.16 cm), followed by T1 (1.99 cm) and T3 (1.98 cm); T4 had the smallest mean (1.77 cm). A similar pattern was observed for tillers; T2 exhibited the largest mean tiller diameter (1.83 cm), T3 and T1 were intermediate (1.63 and 1.62 cm, respectively), and T4 had the lowest mean tiller diameter (1.55 cm). To date, studies on tunicate maize have largely focused on the molecular and genetic bases of alternative reproductive phenotypes associated with the *Tu* and *ts* mutations, which produce abnormal male and female structures [11,12,13,14,15]. By contrast, most non-scientific web sources [18,71,72] offer divulgation-level descriptions that emphasize the distinctive morphology of this race, particularly the E2 cob morphotype [3], without quantitative detail. Consequently, to our knowledge, phenological traits such as plant height, stem diameter, and tiller number have not been systematically addressed as primary research objectives in this corn race.

Although vegetative variables have not previously been quantified in tunicate maize, our findings can be contextualized using reports for other native races. In maize adapted to tropical climates, stem diameters range from 1.35 to 2.0 cm [59,73], whereas in temperate climates with summer rains they span 1.83–2.9 cm [24,25]. These benchmarks are consistent with the environmental conditions of the High Valleys of Mexico, where our field experiments were conducted [58]. Accordingly, the stalk diameters measured here, both main stems and tillers, fall within these reported ranges across all pod corn treatments.

#### 2.3.2. Reproductive Dependent Variables

The results show that in plot P1, significant differences were detected among treatments for yield-dependent variables, including tassel type, kernel morphotypes, tassel grain, and grain yield (Table 4). T1 produced the highest mean number of stems bearing wild-type tassels (84.67), followed by T3 (35.28) and T2 (27.71), whereas T4 had the fewest. Wild-type tassels observed in morphotypes Tp1 and Tp4 were consistent with phenologies reported for commercial maize grown in Mexico and elsewhere [24,25,35,64], with variation largely limited to the number of lateral branches arising from the main spike [74].

Among stems bearing hermaphrodite tassels (Tp2 morphotype), T3 showed the highest mean (52.92), followed closely by T2 (51.48) and T4 (48.60), whereas T1 had the lowest (1.75; Table 4). A few tassel-less stems were observed only in T1; in these cases, the terminal inflorescence was replaced by an ear with fully functional husks. Phenological descriptions for Tp2 and Tp3 remain scarce, as most studies on pod corn have focused on the genetic basis of *Tu*/*ts*-associated phenotypes [7,8,10,11,12,13,14]. In commercial maize, similar expressions are typically classified as abnormal tassels or spikes and are seldom characterized, as such plants are usually removed from the experimental plots [32,57,75].

Ear number per plant differed among treatments (Table 4). T4 showed the highest mean (1.47 ears per plant), followed by T2 (1.33) and T3 (1.19), whereas T1 had the lowest (0.92). In commercial maize, within-row spacing markedly influences ear set, with typical means of 1.05–1.11 ears per plant at 25 and 30 cm, respectively [76]. Overall, the values for T2-T4 are comparable to or higher than those reported for these spacings, while T1 falls below this range.

The ear numbers observed here are consistent with reports for other maize races from the same region (1.04–1.19 ears per plant) [24,25]. Notably, T4 and T2 produced more ears per plant than T3 and T1. This pattern mirrors the tillering response; T2 and T4 exhibited fewer tillers per plant (Table 3), which likely reduced the effective stand density and favored more complete silk pollination (Table 4). By contrast, the higher effective density in T3 and T1, reflected in the greater totals of stems per plot (88.2 and 87.75, respectively), likely intensified intraspecific competition and reduced ear set, a mechanism well documented for maize at elevated planting densities [77,78].

Significant treatment effects were detected for kernel morphotypes and tassel grain (Table 5). In Tp4 plants from T2, T3, and T4, the E4 and E5 ear morphotypes were rare (<1.5% of plants per treatment), yielding too few kernels for independent analysis. To preserve statistical power, kernels from these rare morphotypes were pooled with those from E1 and E2 within each treatment. We applied an a priori classification; kernels from E2 and E4 were assigned to the tunicate group, whereas kernels from E1 and E5 were assigned to the non-tunicate group.

Counts of ears with abundant tunicate kernels (E2/E4) were highest in T2 (55.15), followed by T3 (53.13) and T4 (45.03), whereas T1 showed a minimal mean (1.16) (Table 5). For semi-tunicate ears (E3), T2 and T3 again led (14.11 and 13.67, respectively), with T4 intermediate (12.76) and T1 lowest (1.33). In contrast, non-tunicate ears (E1/E5) were most frequent in T1 (79.56), followed by T4 (41.65), T3 (38.68), and T2 (36.67). To our knowledge, no published studies provide directly comparable distribution for tunicate maize; thus, these results offer a first quantitative baseline for kernel-morphotype frequencies in pod corn. Tassel grain counts ranged from a single grain to > 400 per tassel. Mean tassel grain mass by treatment was as follows: T4, 0.545 kg; T2, 0.405 kg; T3, 0.248 kg; and T1, 0.049 kg (Table 5). As expected, T1, having the fewest hermaphrodite tassels, produced the lowest grain mass. These results agree with prior descriptions of tassel-seed phenotypes, in which tassels commonly set > 100 grains [9,14]. Although earlier studies treat this trait only briefly, the present data provide a quantitative baseline for tassel-grain yield in pod corn. Morphologically, tassel grains arise from pedicellate spikelets (8–10 mm in length) that typically alternate with sessile spikelets; in addition, some branch tips retain staminate flowers, enabling self-pollination [11,13,14].

Grain yield differed significantly among treatments (Table 5). T2 achieved the highest mean yield (10.78 t ha^−1^), followed by T4 (9.68 t ha^−1^) and T3 (9.63 t ha^−1^), whereas T1 produced the lowest (6.32 t ha^−1^). These yields are hectare-standardized estimates obtained by extrapolating total ear grain harvested per treatment (32.04 m^2^; three replicates combined) to a 1 ha basis and correcting to 12% moisture. Overall, yields across pod corn treatments fall within the range reported for hybrid maize (≈5.5 to > 13.5 t ha^−1^) [78,79,80]. To interpret these contrasts, we next examined stand architecture, particularly tillering, and resource use efficiency. Grain yield was significantly higher in T2–T4 than in T1. It has been reported that hybrid maize with 1–4 tillers per plant tends to achieve greater yields than stands with more tillers [65]. However, this trend did not hold for T1; despite averaging 1.95 tillers per plant, plants set fewer than one ear per plant, which likely constrained yield. Because grain production depends on resource availability and uses efficiency [69], the combination of relatively greater vegetative sinks in T1 and suboptimal ear set, together with biotic and abiotic factors, probably depressed final grain yield.

While the yields (t ha^−1^) reported for treatments T2–T4 are comparable to those reported for high-performing hybrid maize and for the upper range of some High Valley landraces, it is important to acknowledge that these values are extrapolations based on measurements from relatively small experimental areas (32.04 m^2^ per treatment within a 153 m^2^ subplot, with buffer alleys). Although standard procedures were applied, extrapolation from small plots may introduce variability (e.g., edge effects and spatial heterogeneity), which can limit the direct transfer of these specific yield estimates to large-scale commercial fields without further validation in larger, multi-location trials.

Compared with the average yields reported for several Mexican High Valley landraces under rainfed management, typically 2.7 to 6.6 t ha^−1^ [24,25,81,82], the values observed for T2–T4 fall at the upper end of the reported range and overlap with the maxima reported for Chalqueño under optimal conditions (8.7–10.5 t ha^−1^) [81]. This comparatively high performance may reflect the combination of (i) intensive organic-mineral fertilization in P1 (bokashi amendment; Section 3.3), (ii) relatively low stand density together with early, synchronized tillering that increased the number of reproductive stems per unit area, and (iii) potential prior gene flow and farmer selection in the source population, which may have incorporated favorable alleles from locally cultivated improved maize [3,4,25]. Nevertheless, given the small plot size, open pollination, and hectare extrapolation, these yield values should be interpreted primarily as plot-scale estimates of productive potential under the tested management rather than as stable, farm-scale expectations; independent validation in larger plots, across seasons, and across locations is warranted.

### 2.4. Proximate Chemical Analysis

The proximate compositions of pod corn kernels with the tunic (T2 ^£^, T3 ^£^, T4 ^£^) and without the tunic (T2 ^€^, T3 ^€^, T4 ^€^) are summarized in Table 6. For moisture, T4 ^£^ showed the highest value (7.21%), exceeding its dehulled counterpart T4 ^€^ (4.03%), while T2 ^£^ and T3 ^£^ did not differ significantly. In samples processed without the tunic (T1, T2 ^€^, and T3 ^€^), moisture also did not differ significantly, ranging from 4.55 to 4.92%. These levels are broadly consistent with reports for native Mexican maize; Esparza et al. [83] documented 8.16%, 9.52%, and 9.09% for Azul México, Azul Oaxaca, and Azul Chihuahua, respectively, and Mex-Álvarez et al. [84] reported 10.43% (purple), 6.86% (red), 7.45% (yellow), 7.46% (white), and 7.98% (hybrid white). In particular, the moisture values for red, yellow, and white creole maize in Mex-Álvarez et al. [84] align with those obtained here for T2 ^£^, T3 ^£^, and T4 ^£^.

Regarding ash content, samples retaining the tunic (T2 ^£^, T3 ^£^, T4 ^£^) did not differ significantly from one another but showed higher values than their dehulled counterparts. In contrast, no differences were detected among dehulled treatments (T1, T2 ^€^, T3 ^€^, T4 ^€^). Reported ash contents for colored maize include 1.7% (purple), 1.29% (red), 1.43% (blue), and 1.84% (orange) [85,86,87]. For native varieties such as Azul México, Azul Oaxaca, and Azul Chihuahua, Esparza et al. [83] reported 1.09%, 1.13%, and 1.16%, respectively; Mex-Álvarez et al. [84] reported 1.42% and 1.39% for native purple and red maize. These values from the literature align with those obtained here for dehulled samples (T1, T2 ^€^, T3 ^€^, T4 ^€^) and are lower than the ash measured in tunic-retaining samples (T2 ^£^, T3 ^£^, T4 ^£^), as expected given the contribution of the tunic. Notably, maize grain composition varies with fertilization [88] and genotype; moreover, the nixtamal flour standard NMX-F-046-S-1980 [89] specifies a maximum ash limit of 1.5% for corn flour [90,91].

Regarding fat content, T2 ^£^ and T3 ^£^ differed significantly from all other treatments. Reported ether extract values for native maize include 6.13% and 5.15% in red and blue varieties, respectively [87]; 4.07% and 5.40% in purple and red varieties [84]; 4.13%, 3.97%, and 4.06% for Azul México, Azul Oaxaca, and Azul Chihuahua [83]; and 3.1% for purple maize [85]. Overall, the fat content observed here is comparable to that reported for blue-grain native maize by Esparza et al. [83].

For crude protein, contents varied among treatments (Table 6); the lowest means were observed in T4 ^£^ (with tunic) and T3 ^€^ (without tunic), whereas the highest were recorded in T1 and T4 ^€^ (without tunic). Values from the literature include 6.76% and 7.45% for purple and red maize [84], and 9.10%, 8.37%, and 9.06% for Azul México, Azul Oaxaca, and Azul Chihuahua [83]. Overall, protein in blue maize ranges from 6.73% to 9.37% [92]. These benchmarks are higher than those obtained here for pod corn kernels. Notably, endosperm type influences protein concentration; crystalline and semi-crystalline maize often exceed dentate or semi-dentate types, although some floury genotypes can also be protein-rich [90], underscoring the role of genotype when developing products that incorporate maize kernels.

Finally, for crude fiber, T1 recorded the highest value relative to the tunicated treatments (T2 ^£^, T3 ^£^, and T4 ^£^). By contrast, those that were non-tunicated (T2 ^€^, T3 ^€^, and T4 ^€^) exhibited the highest fiber content overall. Mex-Álvarez et al. [84] reported crude fiber contents of 3.32% and 6.39% for purple and red native maize, respectively. Other studies on the Xmejen nal (yellow), T’síit bakal (white), and Creole purple (purple) maize landraces reported crude fiber contents of 1.65%, 0.72%, and 1.0%, respectively [85,86]. The crude fiber content observed in this study is similar to that reported by Cázares-Sánchez et al. [86] for purple maize.

## 3. Materials and Methods

### 3.1. The Samples’ Origin

**Figure 7:**
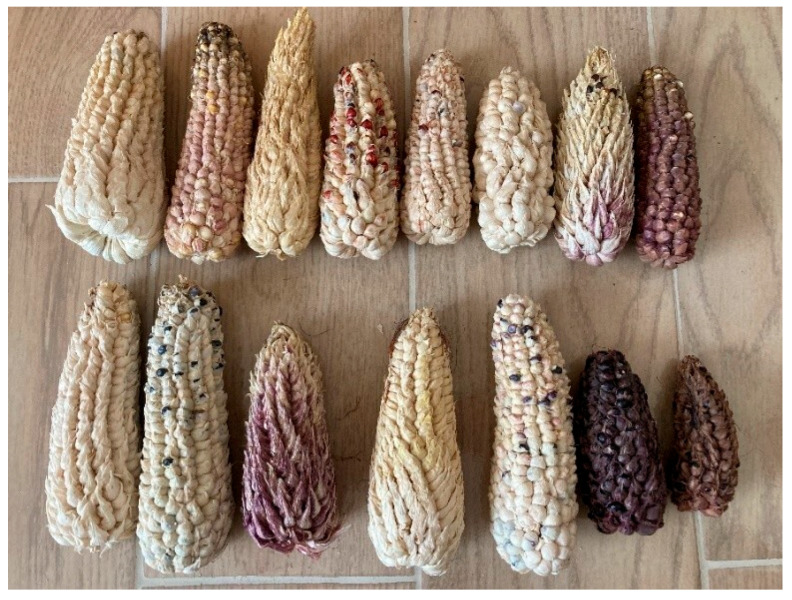
Representative tunicate pod corn ears (*Zea mays* var. *tunicata*).

Field trials for the 2022 crop cycle were established using seeds from tunicate ears collected from local pod corn producers at the 24th Corn and Other Native Seeds Fair in Vicente Guerrero, Españita, Tlaxcala, Mexico, during the 2021 cycle (Figure 7). In 2023–2024, a subsequent experimental plot was sown with seeds produced in 2022 to conduct individual phenological evaluations of plant morphotypes (including wild-type and abnormal male inflorescences) and ear types (no tunic, abundant tunic, scarce tunic), and to quantify tassel-derived grain. Plant material for this phase originated from the preliminary plots P1 and P2. For the 2023–2024 evaluation, seed was organized into four composite lots by pooling grain from multiple parental plants/ears or tassels that expressed the corresponding phenotype in the 2022 preliminary plots (P1 and P2). Because these treatments were not structured progenies (half-sib/full-sib families) and were produced under open pollination, the study is intended for phenotypic mean comparisons and baseline characterization rather than estimation of heritability, genetic variance, or genetic gain.

### 3.2. Population Definition and Scope

The material evaluated corresponds to a composite sample of a locally maintained pod corn landrace sourced from producers in the Yumhu community (San Juan Ixtenco, Tlaxcala, Mexico). Because the study did not include genotyping, controlled crosses, or family-structured progenies, we did not test the Hardy–Weinberg equilibrium or quantify population genetic structure; instead, we compared phenotypic means among phenotype-derived seed lots (T1–T4) to establish a quantitative baseline for future breeding, conservation, and population-genetic work.

### 3.3. Plot Location and Experimental Design

During the 2022 spring–summer cycle in the High Valleys of Mexico, two preliminary rainfed experimental plots (32 m^2^ each) were established in Apan, Hidalgo (19°65′48″ N, 98°51′88″ W), and San Dionisio Yauhquemehcan, Tlaxcala (19°41′21″ N, 98°18′21″ W). The plots were sown to evaluate phenological traits of tunicate ears from pod corn sourced from local producers in the Yumhu community of San Juan Ixtenco, Tlaxcala. Both sites have a temperate subhumid climate with summer rains, and the dominant soil types are Regosol, Phaeozem, and Cambisol [58,93,94]. Each plot comprised nine rows, 4 m long and 0.8 m apart. One seed was sown every 26.67 cm within the row, for a target density of 42,188 plants per ha. Each plot contained a maximum of 135 plants, arranged in three subplots that served as experimental replicates (Table 7). Crop management followed recommendations for maize in the High Valleys of Mexico [24]. Recorded variables included plant height, number of tillers per plant [17], and grain yield corrected to 12% moisture, expressed in t ha^−1^ [95].

**Figure 8:**
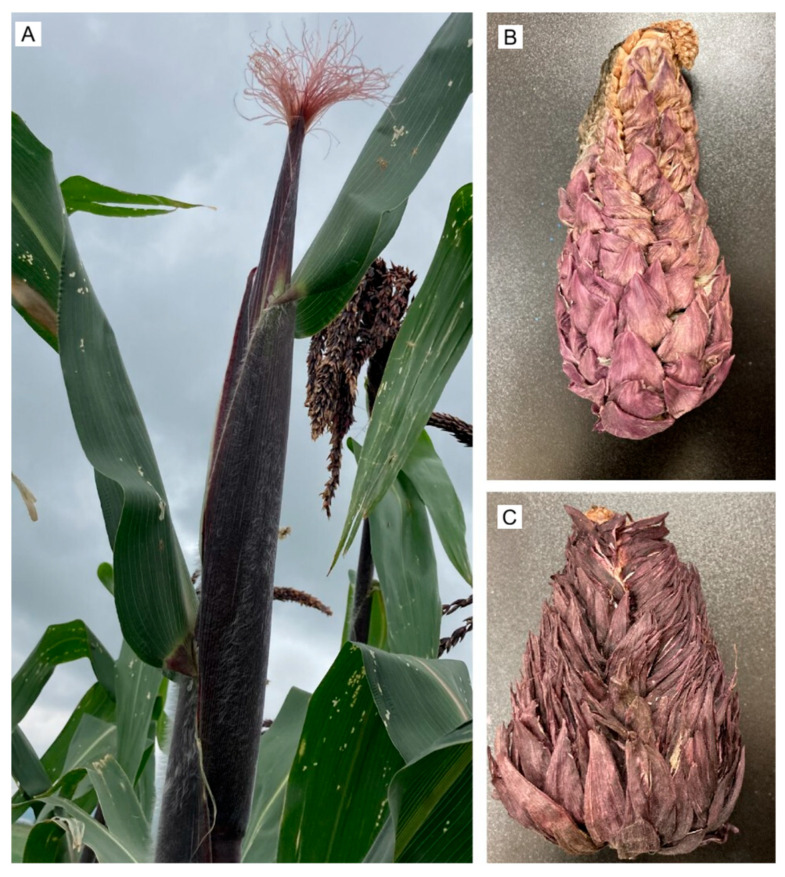
Female plant bearing a tunicate ear. (**A**) Plant with a developing ear. (**B**) Tunicate ear with some viable kernels. (**C**) Poorly developed tunicate ear lacking grains.

Building on the results from the preliminary plots (Table 7), a dedicated subplot within P1 was established to evaluate the phenology of pod corn ears and tassels during the 2023–2024 crop cycle (Table 8). In the 2022 cycle, a mean of 2.33 ± 0.57 and 1.33 ± 0.57 plants per replicate in P1 and P2, respectively, failed to produce a tassel and instead formed a tunicate ear at the stalk apex (Figure 8). These atypical plants were excluded from the 2023–2024 treatments because most produced sterile ears; in the few cases with grain set, yields were insufficient for subsequent analyses.

Following Han et al. [11], pod corn ear phenotypes were classified as (i) wild-type (grains not covered by a tunic); (ii) heterozygous *Tu* ears, with glumes partially elongated relative to wild-type; and (iii) homozygous *Tu*, with fully elongated glumes and abnormal, arrow-shaped branches. Tassel mutant (heterozygous and homozygous *Tu*) were analyzed jointly and referred to as hermaphrodite tassels, as our assessments emphasized morphological and phenological traits rather than genotyping. Under open-pollinated field conditions, the source of pollen fertilizing hermaphrodite tassels could not be determined with confidence because wind and gravity likely mediated pollen transfer among treatments [96]. This sampling intensity is adequate for detecting differences in treatment means for continuous traits. Still, it was not designed to resolve the segregation ratios or estimate quantitative genetic parameters, which would require family-based progeny arrays and/or genotyping.

**Figure 9:**
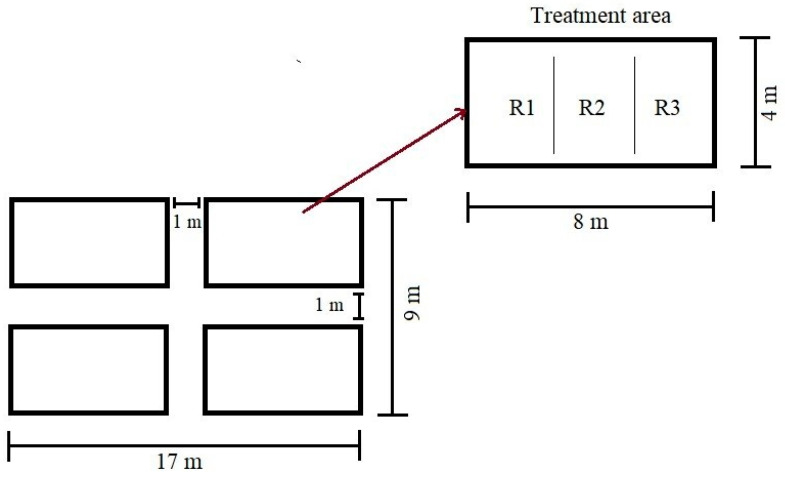
Treatment setup within the P1 experimental plot R1, R2, and R3 indicate the replicates within each treatment).

The P1 experimental plot for the 2023–2024 cycles covered 153 m^2^ (9 × 17 m). Treatments were arranged in a completely randomized design with three replicates (Figure 9). To limit cross-pollination among treatments, 1 m unplanted buffers were maintained between treatment blocks [24]. Each replicate comprised three 4 m rows spaced 0.89 m apart. Within each row, 15 hills were established at 26.67 cm intervals, sowing three seeds per hill (45 seeds per row). At ≈30 days after emergence (V8–V10), stands were thinned to one vigorous plant per hill, yielding 15 plants per row (45 per replicate; 135 per treatment) for phenological assessment [97].

A completely randomized design (CRD) was selected for the P1 phenology trial because treatments were defined by seed lots derived from contrasting ear/tassel phenotypes, and the crop was grown under open-pollinated conditions. To minimize cross-pollination and admixture among treatments, unplanted buffer alleys (1 m) were maintained between treatment plots. Importantly, these buffers were used for isolation purposes; therefore, the experiment was not established nor analyzed as a randomized complete block design (RCBD). The trial was implemented within a relatively small (153 m^2^) P1 subplot under uniform agronomic management, using randomized treatment allocation, three replicates per treatment, and a standardized plant density (thinned to one vigorous plant per hill) to help mitigate residual micro-site variability.

The P1 plot was fertilized with a bokashi-type fermented amendment (Table 9). In 2022, 20% of the total dose (1000.35 kg) was applied; the remaining 80% (4001.4 kg) was distributed across the 2023–2024 cycles (2000.7 kg per year). Each cycle included two applications: (i) pre-sowing during soil preparation and (ii) at 60–65 days of vegetative growth, applying half of the seasonal dose at each timing, for a total of 7.41 kg per plant.

For P2, fertilization in 2022 was conducted in two stages (Table 10), with 20% of the dose (544 kg) applied before sowing during land preparation as cow manure at an average rate of 10 kg per m^2^ and the remaining 80% (2176 kg) applied 65 days after the onset of vegetative growth at ≈7 kg per m^2^ using a mixture of cow manure-based compost, corn straw, coal cinders, activated mountain microorganisms, and rock powders.

Land preparation in plots P1 and P2 consisted of plowing followed by cross-tilling. Sowing was carried out from May to October during 2022–2024 [93]. All experimental plots were managed uniformly across a six-month crop cycle. At the end of each cycle, selected growth and yield parameters were measured at the vegetative development stage (70–90 days) and at maturation (150–180 days) [24]. During the vegetative stage, plant height (main stem and tillers), number of tillers per plant, and stalk diameter (measured 10 cm above the ground for both the main stem and tillers) were recorded [17].

During the reproductive stage in plot P1, we recorded tassel phenotype (wild-type, hermaphroditic, and tassel-less), ear morphotypes, and grain yield. Grain yield was standardized to 12% moisture and expressed as t ha^−1^, calculated from a harvested treatment area (32.04 m^2^ per treatment; 0.003204 ha) and converted to a hectare basis [95].

### 3.4. Proximate Composition of Grain

Biochemical analyses were performed on pod corn using composite samples prepared by pooling kernels from the central portion of five ears per ear morphotype within each treatment, following Galicia et al. [98]. Kernels from each treatment were ground in a cyclone mill (Udy Corporation, TecnoCientífica^®^, Buenos Aires, Argentina; Model 3010-019) and passed through a 35-mesh (0.5 mm) metal sieve to obtain a uniform flour. Corn flour samples, reagents, and other materials were weighed on an analytical balance (OHAUS^®^, Tlajomulco de Zúñiga, Jalisco, Mexico; Model DV314C). The proximate composition of kernels from each ear morphotype and tassel-derived grains across the four pod corn treatments was determined according to AOAC Official Methods as being 923.03, 920.35, 925.10, and 991.20 [99], for ash, fat, moisture, fiber, and protein, respectively.

### 3.5. Statistical Analysis

The vegetative and reproductive variables of pod corn were evaluated during August–September and October–November of 2022, 2023, and 2024. The experiment followed a completely randomized design with three replicates. For the 2023–2024 phenology trial (P1), treatment (T1–T4; phenotype-defined seed lots) was analyzed as a fixed effect, whereas replicate (subplot) was treated as a random effect (experimental unit). Comparisons between the two preliminary plots (P1 vs. P2, 2022) are reported and discussed descriptively because plot differences are confounded with location and management (fertilization regime) and were not independently manipulated. Before analysis, the homogeneity of variances was assessed with Bartlett’s test [17]. Continuous response variables (e.g., plant height, stalk diameter, ear length, grain yield, and proximate composition percentages) were analyzed using linear models with Gaussian (normal) errors (ANOVA) in SAS, version 15.3. Count-type variables (e.g., numbers of tillers and ears per plant, and frequencies of tassel/ear phenotypes) were analyzed using generalized linear (mixed) models with an appropriate-count distribution (Poisson or, when overdispersion was detected, negative binomial). When overall effects were significant, means were compared using Tukey’s HSD at *p* < 0.05. All statistical analyses were performed with SAS-STAT [100]. To support pooling across the 2023–2024 cycles and within-treatment morphotypes, year (2023 vs. 2024) and plant morphotype (Tp1–Tp4) were included in preliminary models. Neither year nor morphotype effects (including their interactions with treatments, where applicable) were significant for any response variable (*p* > 0.05); therefore, data were pooled across years, and morphotypes were averaged within treatments for subsequent analysis.

## 4. Conclusions

Tunicate maize is locally produced by producers in the Yumhu community in Tlaxcala, Mexico. Pod corn has received limited scientific attention and remains unfamiliar to the general public. Which is why it was important to characterize morphology, phenology, and proximate composition, phenotypic performance, and variability among phenotype-derived seed lots under the evaluated field conditions.

The results showed that significant treatment effects were observed for vegetative and reproductive traits. In particular, the T2 showed the most significant plant height (main stem: 250.74 cm; tillers: 241.55 cm) and the highest grain yield (10.78 t ha^−1^), the T3 presented the highest tiller number (1.96 tillers plant^−1^), while T4 produced the highest number of ears per plant (1.47 ears plant^−1^). Kernel morphotype frequencies differed markedly among treatments; ears with abundant tunicate kernels (E2/E4) were most frequent in T2 (55.15) and T3 (53.13), whereas non-tunicate ears (E1/E5) predominated in T1 (79.56).

Finally, proximate composition analysis showed that the presence of the tunic significantly affects grain chemistry. Compared with dehulled samples (T ^€^), tunic-retaining samples (T ^£^) consistently showed higher moisture and ash contents (e.g., T2 ^£^: 6.53% moisture and 3.07% ash vs. T2 ^€^: 4.92% and 1.55%). In contrast, dehulled samples tended to show higher fiber (e.g., T2 ^€^: 1.77% vs. T2 ^£^: 0.96%) and higher fat in T2 and T3, while protein responses were treatment-dependent (e.g., higher in T2 ^€^ and T4 ^€^ than in T2 ^£^ and T4 ^£^, but lower in T3 ^€^ than in T3 ^£^). Collectively, these results provide data-driven reference values for agronomic performance and grain composition of tunicate maize under the evaluated conditions and support its characterization for potential food-related applications.

## Figures and Tables

**Table 1:** Vegetative variables of pod corn plants derived from tunicate corn ears.

Key **	Year	Plant Height ± SE *	Tillers Per Plant ± SE *	Stalk Diameter ± SE *
P1	2022	264.24 ± 1.66 a	2.99 ± 0.03 a	2.91 ± 0.09 a
P2	2022	257.89 ± 0.15 b	2.56 ± 0.02 b	2.90 ± 0.03 a

* Means with the same letter in the same column do not differ significantly (*p* < 0.05, Tukey-adjusted multiple comparisons), ± standard error (SE). ** P1 = Plot 1 (Apan, Hidalgo, Mexico); P2 = Plot 2 (Yauhquemehcan, Tlaxcala, Mexico).

**Table 2:** Reproductive variables of pod corn plants derived from tunicate corn ears.

Key **	Year	Ears Per Plant ± SE *	Normal Tassel ± SE *	Hermaphrodite Tassel ± SE *	Plant Without Tassel ± SE *	Grain Yield ^€^ t ha^−1^ ± SE *
P1	2022	2.59 ± 0.05 a	40.33 ± 0.57 b	91.33 ± 1.15 a	2.33 ± 0.57 a	12.23 ± 0.21 a
P2	2022	2.06 ± 0.06 b	59.67 ± 0.57 a	56.00 ± 0.90 b	1.33 ± 0.57 b	11.91 ± 0.16 a

* Values bearing the same letter within a column do not differ significantly (*p* < 0.05; Tukey-adjusted multiple comparisons), ± standard error (SE). ^€^ Grain yield was calculated as the sum of the weights of all grains produced by each ear type (abundant tunic, scarce tunic, no tunic) and by the hermaphrodite tassel; where present, tunics were removed before weighing. The resulting sum represents the final total weight per treatment. ** P1 = Plot 1 (Apan, Hidalgo, Mexico); P2 = Plot 2 (Yauhquemehcan, Tlaxcala, Mexico).

**Table 3:** Vegetative variables of pod corn plants.

Key ^¥^	Plant Height ± SE *		No. of Tillers Per Plant ± SE *	Stalk Diameter ± SE *	
	Main Stem **	Tillers **		Main Stem **	Tillers **
T1	210.41 ± 0.28 b	192.24 ± 0.24 b	1.95 ± 0.01 a	1.99 ± 0.01 b	1.62 ± 0.01 b
T2	250.74 ± 0.41 a	241.55 ± 0.51 a	1.76 ± 0.02 b	2.16 ± 0.02 a	1.83 ± 0.02 a
T3	210.61 ± 0.27 b	192.12 ± 0.49 b	1.96 ± 0.03 a	1.98 ± 0.02 b	1.63 ± 0.03 b
T4	187.25 ± 0.96 c	168.66 ± 0.41 c	1.50 ± 0.02 c	1.77 ± 0.02 c	1.55 ± 0.02 c

* Means sharing the same letter within a column are not significantly different (*p* < 0.05; Tukey-adjusted multiple comparisons), ± standard error (SE). ** Variables are expressed in centimeters (cm). ^¥^ T1 = grain from wild-type ears (no tunic) of plants with wild-type tassels; T2 = grain from ears with an abundant tunic of plants with a hermaphrodite tassel; T3 = grain from ears with scarce tunic of plants with hermaphrodite tassels; T4 = grain from the hermaphrodite tassel.

**Table 4:** Tassel type and number of ears produced per plant within each treatment.

Key **	Tassel Type			No. of Ears Per Plant ± SE *
	Wild ^€^ ± SE *	Hermaphrodite ^€^ ± SE *	Without Tassel ^€^ ± SE *	
T1	84.67 ± 0.15 a	1.75 ± 0.01 d	1.33 ± 0.58 a	0.92 ± 0.01 d
T2	27.71 ± 0.45 c	51.48 ± 0.09 b	0.00 ± 0.00 b	1.33 ± 0.01 b
T3	35.28 ± 0.61 b	52.92 ± 0.81 a	0.00 ± 0.00 b	1.19 ± 0.02 c
T4	18.90 ± 0.54 d	48.6 ± 0.05 c	0.00 ± 0.00 b	1.47 ± 0.01 a

^€^ Total number of plants with the same tassel type within each treatment. * Means sharing the same letter within a column are not significantly different (*p* < 0.05; Tukey-adjusted multiple comparisons), ± standard error (SE). ** T1 = grain from wild-type ears (no tunic) of plants with wild-type tassels; T2 = grain from ears with an abundant tunic of plants with a hermaphrodite tassel; T3 = grain from ears with scarce tunic of plants with hermaphrodite tassels; T4 = grain from the hermaphrodite tassel.

**Table 5:** Different kernel morphotypes, tassel grain, and grain yield by each treatment.

Key ^£^	Tunicate ^€^ ± SE *	Semi-Tunicate ^€^ ± SE *	Without Tunic ^€^ ± SE *	Tassel Grain (kg) ^¥^ ± SE *	Grain Yield ** t ha^−1^ ± SE *
T1	1.16 ± 0.40 d	1.33 ± 0.51 c	79.56 ± 0.22 a	0.049 ± 0.010 d	6.32 ± 0.02 c
T2	55.15 ± 0.98 a	14.11 ± 0.30 a	36.67 ± 0.34 d	0.405 ± 0.005 b	10.78 ± 0.05 a
T3	53.13 ± 0.52 b	13.67 ± 0.34 a	38.68 ± 0.11 c	0.248 ± 0.015 c	9.63 ± 0.08 b
T4	45.03 ± 0.06 c	12.76 ± 0.08 b	41.65 ± 0.19 b	0.545 ± 0.003 a	9.68 ± 0.01 b

* Means sharing the same letter within a column are not significantly different (*p* < 0.05; Tukey-adjusted multiple comparisons), ± standard error (SE). ^€^ Mean number of ears per ear morphotype (E1–E5) within each treatment. ^¥^ Mean total grain weight (kg) produced by the hermaphrodite tassel per treatment (range: 1–408 grains per tassel). ** Sum of grains by all cobs across ear morphotypes E1–E5 treatment. ^£^ T1 = grain from wild-type ears (no tunic) of plants with wild-type tassels; T2 = grain from ears with an abundant tunic of plants with a hermaphrodite tassel; T3 = grain from ears with scarce tunic of plants with hermaphrodite tassels; T4 = grain from the hermaphrodite tassel.

**Table 6:** Proximate chemical analysis of the tunicate corn grains.

Key *	Moisture % ± SE ^1^	Ash % ± SE ^1^	Fat % ± SE ^1^	Protein % ± SE ^1^	Fiber % ± SE ^1^
T1	4.55 ± 0.03 c	1.46 ± 0.09 c	4.65 ± 0.03 ab	6.52 ± 0.24 a	1.26 ± 0.11 c
T2 ^£^	6.53 ± 0.08 b	3.07 ± 0.23 a	3.81 ± 0.29 c	3.78 ± 0.19 bc	0.96 ± 0.02 d
T3 ^£^	6.68 ± 0.08 b	3.03 ± 0.33 a	3.61 ± 0.28 c	3.57 ± 0.44 c	0.90 ± 0.05 de
T4 ^£^	7.21 ± 0.06 a	2.58 ± 0.48 ab	4.69 ± 0.12 ab	2.15 ± 0.39 d	0.78 ± 0.01 e
T2 ^€^	4.92 ± 0.44 c	1.55 ± 0.42 c	5.08 ± 0.07 a	4.61 ± 0.51 b	1.77 ± 0.07 a
T3 ^€^	4.83 ± 0.03 c	1.12 ± 0.12 c	4.87 ± 0.25 ab	1.05 ± 0.43 e	1.59 ± 0.06 b
T4 ^€^	4.03 ± 0.12 d	1.80 ± 0.38 bc	4.45 ± 0.16 b	6.64 ± 0.23 a	1.65 ± 0.07 ab

^1^ Standard error (SE). Within each column, means followed by different letters differ significantly (*p* < 0.05). ^£^ Treatments in which grains were ground together with their tunic. ^€^ Treatments in which the tunic was removed from the grains before grinding. * T1 = grain from wild-type ears (no tunic) of plants with wild-type tassels; T2 = abundant-tunic ears from plants with hermaphrodite tassels; T3 = scarce-tunic ears from plants with hermaphrodite tassels; T4 = grain from hermaphrodite tassels.

**Table 7:** Characteristics of the experimental plots conducted with tunicate ears.

Key ^£^	Treatments	Location	AAPT *	Masl **
P1	Plot 1	Escuela Superior de Apan-UAEH	622 mm, 14.1 °C	2490
P2	Plot 2	El Terregal de Ray	795 mm, 16.5 °C	2420

* Annual average precipitation and temperature. ** Meters above sea level. ^£^ P1 = Apan, Hidalgo, Mexico; P2 = Yauhquemehcan, Tlaxcala, Mexico.

**Table 8:** Treatments used to assess phenological characteristics by ear type.

Key	Treatments
T1	Grain from wild-type ears (no tunic) of plants with wild-type tassel
T2	Grain from ears with an abundant tunic of plants with a hermaphrodite tassel *
T3	Grain from ears with scarce tunic of plants with hermaphrodite tassels *
T4	Grain from the hermaphrodite tassel *

* For these treatments, plants with tassels expressing the *Tunicate* (*Tu*) mutation were analyzed jointly: heterozygous *Tu* plants with partially elongated, feminized florets and homozygous *Tu* plants with fully elongated, feminized florets [11]. Seed for each treatment represents a pooled (bulked) lot from multiple parental plants expressing the target ear/tassel phenotype in the preliminary plots, to improve the representativeness of the sampled landrace while maintaining the phenotype-defined grouping.

**Table 9:** Bokashi fermentation process used in plot P1.

Ingredients	Amount (kg)
Schinus molle brushwood	6.64
Corn grain with bioinoculants ^a^	97.64
Lake-bottom sediments ^b^	27.95
Agricultural lime	10.91
Black topsoil	931.12
Tepetate	502.76
Vermicompost	140.18
Tobacco dust	1.63
Sawdust	19.66
Peat moss	1.09
Coffee grounds	86.60
Ground eggshell	1.93
Sheep manure	1065.66
Dog manure	224.70
Medium-chopped corn stubble	1154.14
Charcoal fines	489.12
Ground coconut coir	0.70
Teosinte stubble (*Zea mays* subsp. *mexicana*)	69.87
Rock powders ^c^	161.76
Poplar brushwood	7.69

^a^ Microorganisms inoculated into the bokashi: *Trichoderma harzianum* (1 × 10^9^ CFU g^−1^); *Arthrobacter globiformis* (27.8 CFU g ^−1^); *Azotobacter chroococcum* (27.8 CFU g^−1^); *Azospirillum lipoferum* (27.8 CFU g^−1^); *Glomus intraradices* (5.05 CFU g^−1^); *G. etunicatum* (0.05 CFU g^−1^); *G. fasiculatum* (5.0 CFU g^−1^); *Pisolithus tinctorius* (649.7 CFU g^−1^); *Scleroderma citrinum* (43.9 CFU g^−1^); *S. geaster* (43.9 CFU g^−1^); *Laccaria laccata* (43.9 CFU g^−1^); *L. bicolor* (43.9 CFU g^−1^); *Nitrospumilus maritimus* (52,203.9 CFU g^−1^); *Azospirillum brasiliense* (5 × 10^4^ CFU g^−1^); *Pseudomonas flourescens* (5 × 10^4^ CFU g^−1^); *Bacillus musilaginosus* (5 × 10^4^ CFU g^−1^); and *B. subtilis* (1 × 10^9^ CFU g^−1^). ^b^ Organic matter derived from filamentous algae, phytoplankton, zooplankton, and remains of aquatic plants like *Typha latifolia*. ^c^ The minerals used were magnesite (6.98 kg), zeolite (6.98 kg), dolomite (6.98 kg), phosphate rock (6.98 kg), leonardite (6.98 kg), potassic red clay (6.98 kg), diatomite (6.98 kg), limestone (56.94 kg), wood ash (6.98 kg), and a blend of natural minerals from Minutrer^®^ (42 kg) and Micrafol^®^ (6.98 kg), providing the following macro and micronutrients (kg per batch): N (2.81); P (3.61); K (1.34); Ca (9.31); Mg (3.53); S (0.35); Si (20.98); Al (3.64); Fe (1.12); Na (0.09); Mn (0.06); Zn (2.12); B (0.0000077); Cu (0.059); Mo (0.0001); Ni (0.0073); and Co (0.0001).

**Table 10:** Compost-type fertilizer used in plot P2.

Ingredients	Amount (kg)
Cow manure	1600.00
Compost ^a^	1117.76
Rock dust ^b^	2.24

^a^ Cow manure (502.99 kg), ground corn stover (502.99 kg), charcoal fines (“cisco”) (111.78 kg), and activated mountain microorganisms (1 × 10^9^ CFU g^−1^). ^b^ Magnesite (0.124 kg), zeolite (0.124 kg), dolomite (0.124 kg), phosphate rock (0.124 kg), leonardite (0.124 kg), potassic clay (0.124 kg), diatomite (0.124 kg), calcined bone ash (0.124 kg), wood ash (0.124 kg), and a mix of natural minerals from Minutrer^®^ (0.96 kg) and Micrafol^®^ (0.164 kg) were used, providing the following macro and micronutrients (kg per batch): N (0.04); P (0.30); K (0.06); Ca (0.50); Mg (0.21); S (0.008); Si (0.89); Al (0.18); Fe (0.04); Na (0.006); Mn (0.001); Zn (0.003); B (0.003); Cu (0.0001); Mo (0.000002); Ni (0.0005); and Co (0.000006).

## Data Availability

All data derived from this research that support the presented results are available from the corresponding author upon reasonable request.

## Abbreviations

The following abbreviations are used in this manuscript:

PCD	Programmed cell death
*ts*	Tasselseed
*Tu*	Tunicate
*ZMM19*	MADS-box developmental regulator
SE	Standard error
t	Ton
ha	Hectare
AATP	Annual average precipitation and temperature
masl	Meters above sea level
m	Meter
cm	Centimeter
kg	Kilogram
mm	Millimeter
UAEH	Universidad Autónoma del Estado de Hidalgo
ESAp	Escuela Superior de Apan
